# Prediction of Radiosensitivity in Head and Neck Squamous Cell Carcinoma Based on Multiple Omics Data

**DOI:** 10.3389/fgene.2020.00960

**Published:** 2020-08-18

**Authors:** Jie Liu, Mengmeng Han, Zhenyu Yue, Chao Dong, Pengbo Wen, Guoping Zhao, Lijun Wu, Junfeng Xia, Yannan Bin

**Affiliations:** ^1^Key Laboratory of Intelligent Computing and Signal Processing of Ministry of Education, Institutes of Physical Science and Information Technology, Anhui University, Hefei, China; ^2^Anhui Provincial Engineering Laboratory of Beidou Precision Agricultural Information, Anhui Agricultural University, Hefei, China; ^3^School of Information and Computer, Anhui Agricultural University, Hefei, China; ^4^Key Laboratory of High Magnetic Field and Ion Beam Physical Biology, Hefei Institutes of Physical Science, Chinese Academy of Sciences, Hefei, China; ^5^Department of Bioinformatics, School of Medical Informatics, Xuzhou Medical University, Xuzhou, China

**Keywords:** head and neck squamous cell carcinoma, radiotherapy, multiple omics data, radiosensitivity, gene signature

## Abstract

Head and neck squamous cell carcinoma (HNSCC) is a malignant tumor. Radiotherapy (RT) is an important treatment for HNSCC, but not all patients derive survival benefit from RT due to the individual differences on radiosensitivity. A prediction model of radiosensitivity based on multiple omics data might solve this problem. Compared with single omics data, multiple omics data can illuminate more systematical associations between complex molecular characteristics and cancer phenotypes. In this study, we obtained 122 differential expression genes by analyzing the gene expression data of HNSCC patients with RT (*N* = 287) and without RT (*N* = 189) downloaded from The Cancer Genome Atlas. Then, HNSCC patients with RT were randomly divided into a training set (*N* = 149) and a test set (*N* = 138). Finally, we combined multiple omics data of 122 differential genes with clinical outcomes on the training set to establish a 12-gene signature by two-stage regularization and multivariable Cox regression models. Using the median score of the 12-gene signature on the training set as the cutoff value, the patients were divided into the high- and low-score groups. The analysis revealed that patients in the low-score group had higher radiosensitivity and would benefit from RT. Furthermore, we developed a nomogram to predict the overall survival of HNSCC patients with RT. We compared the prognostic value of 12-gene signature with those of the gene signatures based on single omics data. It suggested that the 12-gene signature based on multiple omics data achieved the best ability for predicting radiosensitivity. In conclusion, the proposed 12-gene signature is a promising biomarker for estimating the RT options in HNSCC patients.

## Introduction

Head and neck squamous cell carcinoma (HNSCC) is the sixth most common malignancy in the world, and nearly 60% of newly diagnosed HNSCC is locally advanced disease (Alsahafi et al., 2019; van der Heijden et al., 2019; Wang et al., 2019). Radiotherapy (RT) is a commonly used adjuvant therapy for HNSCC in addition to surgical treatment (Jemal et al., 2011; Suh et al., 2015). But each of HNSCC patients receiving the same dose of RT has different responses due to the complexity and heterogeneity of tumor, and some patients even have RT injury and secondary cancer (Scaife et al., 2015). Globally, the prognosis of HNSCC patients receiving RT remains a challenge. Therefore, prognostic biomarkers of radiosensitivity prediction for HNSCC are needed to improve RT options and predict treatment response.

In recent years, several studies have suggested that miRNAs, lncRNAs, and some of their target genes were correlated with the RT outcomes in HNSCC patients (Leucci et al., 2016; Weng et al., 2016; Chen et al., 2018; Han et al., 2018). For instance, the upregulation of miR-494-3p expression can enhance the radiosensitivity of HNSCC (Weng et al., 2016), and the upregulation of LINC00473 promotes the radioresistance of HNSCC cells (Han et al., 2018). Furthermore, some gene expression-based signatures have been constructed to predict the survival rate of HNSCC patients with RT. For example, Eschrich et al. (2009) developed the radiation sensitivity index, which was used to predict survival probability in HNSCC patients receiving concurrent chemoradiotherapy. Ma et al. (2019) identified a 4-gene methylation signature to predict the survival rate of HNSCC patients with RT. However, these studies only used single omics data which could not draw more comprehensive associations between complex molecular characteristics and cancer phenotypes. By contrast, multiple omics data involve multidimensional studies of cancer cells, potentially revealing the molecular mechanisms behind different phenotypes of cancer, such as metastasis and recurrence (Chakraborty et al., 2018; Xi et al., 2018; Wang et al., 2020). Therefore, a model based on multiple omics data could be an effective method for radiosensitivity prediction of HNSCC patients.

In this work, in order to construct reliable biomarkers for predicting RT response in HNSCC, we used the gene expression data and copy number variation (CNV)/single nucleotide variation (SNV) data from The Cancer Genome Atlas (TCGA) (Tomczak et al., 2015) to develop a gene signature by the two-stage regularization (2SR) (Lin et al., 2015; Hu et al., 2019) and multivariable Cox regression (Gui and Li, 2005; Benner et al., 2010) models. Then, we evaluated the ability of the gene signature for predicting radiosensitivity by Kaplan–Meier survival analysis (Tripepi and Catalano, 2004). Furthermore, we constructed a nomogram based on the gene signature and clinical variables to facilitate a more intuitive prediction of 3-year and 5-year survival rates for HNSCC patients receiving RT.

## Materials and Methods

**FIGURE 1:**
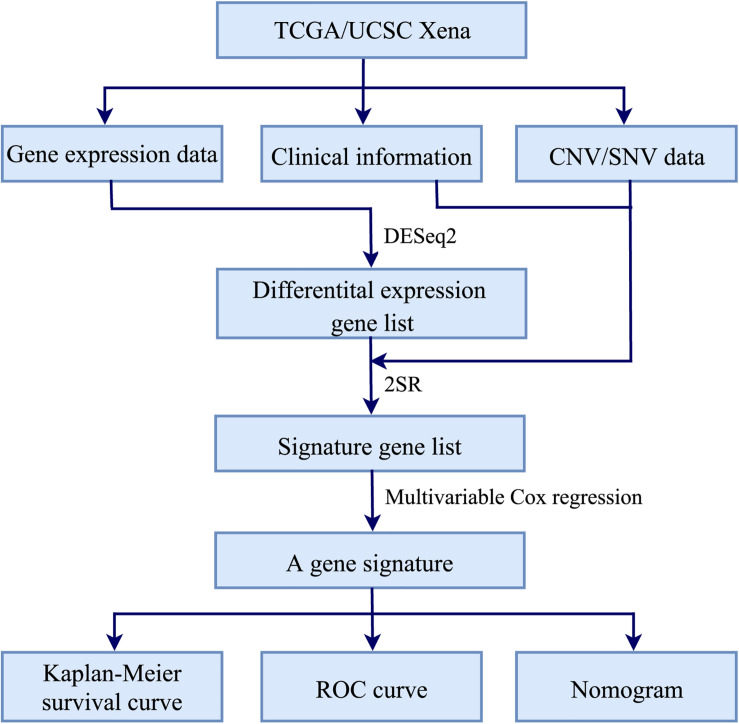
Workflow of constructing a gene signature for predicting RT response in HNSCC.

Figure 1 illustrated the workflow of the proposed signature for predicting RT response in HNSCC. Firstly, R package DESeq2 (Love et al., 2014) was used to identify differential expression genes (DEGs) between HNSCC patients receiving RT and without RT. Secondly, the 2SR and multivariable Cox regression models were used to construct a gene signature associated with the radiosensitivity prediction of HNSCC patients. Finally, Kaplan–Meier survival analysis and time-dependent receiver operating characteristic (ROC) curves (Heagerty et al., 2000; Kamarudin et al., 2017) were used to evaluate the performance of the gene signature. And a nomogram based on the gene signature and clinical variables was constructed to predict the 3-year and 5-year survival rates. The major procedures were described in the following sections.

### Data Processing

We downloaded the transcriptomic gene expression data and the clinical follow-up data of HNSCC patients from TCGA (Tomczak et al., 2015). Meanwhile, we collected the genomic CNV/SNV data from UCSC Xena platform (Goldman et al., 2019). Firstly, we abandoned samples with overall survival (OS) less than 30 days to avoid the impact of deaths with unrelated causes (Chen et al., 2018), and a total of 476 HNSCC patients were analyzed. Furthermore, the available clinical variables included gender, age, OS, vital status, T stage, N stage, clinical stage, tumor grade, and RT options. Secondly, we obtained 20,557 common genes from the gene expression data and the CNV/SNV data, which were used to screen the gene signature associated with the radiosensitivity prediction of HNSCC in subsequent analysis. Thirdly, DESeq2 was used in the normalization of gene expression data and the detection of DEGs between 287 HNSCC patients with RT and 189 patients without RT. As a consequence, genes with |log_2_ fold change| ≥ 1 and false discovery rate (FDR) < 0.01 were defined as DEGs for the further analysis. Finally, the CNV/SNV data of DEGs were converted into a sample-by-gene matrix. If there were one or more mutations within the gene for each sample, the gene-level mutation status value in sample-by-gene matrix was defined as 1; otherwise, it was defined as 0.

### Establishment of the Gene Signature

Based on DEGs, we used the 2SR and multivariable Cox regression models to construct a gene signature that can predict the radiosensitivity of HNSCC patients. The 2SR model could integrate multiple layer omics data to identify signature genes. Specifically, on the first layer, we predicted gene expression values using the CNV/SNV data of DEGs. On the second layer, we used a regularization methodology to regress the predicted gene expression on the first layer and performed signature genes selection and estimation. Multivariable Cox regression model was used to estimate regression coefficients for the identified signature genes. From this model, gene score of HNSCC patients was described as the sum of the products of individual gene expression levels and the estimated regression coefficients. The detailed processes were elaborated in the following.

Firstly, the 287 patients with RT were randomly divided into a training set (*N* = 149) and a test set (*N* = 138) (Table 1). Secondly, on the training set, we input gene expression data, clinical data, and the sample-by-gene matrix of DEGs into the 2SR model to select the OS-related signature genes. The 2SR model was used with default parameters, and the output of this model was a list of genes and their correlated coefficients with OS in patients with RT. When the correlation between genes and OS reached 80% and above, these genes were identified as signature genes. Finally, we calculated the coefficients of these signature genes using multivariable Cox regression model and constructed a gene signature according to the expression levels of these genes, which can stratify HNSCC patients into the high- and low-score groups with the median score of the gene signature on the training set as cutoff value.

**TABLE 1:** Clinical variables of the 287 HNSCC patients with RT.

Variable	Subgroup	Total		Training set		Test set		P*
		*N*	%	*N*	%	*N*	%	
Age (year)	≤60	156	54.36	87	58.39	69	50.0	0.22
	>60	131	45.64	62	41.61	69	50.0	
Gender	Male	224	78.05	123	82.55	101	73.19	0.15
	Female	63	21.95	26	17.45	37	26.81	
T stage	1	14	4.88	9	6.04	5	3.62	0.34
	2	66	22.30	27	18.12	39	28.26	
	3	79	27.53	45	30.20	34	24.64	
	4	122	42.51	63	42.28	59	42.75	
N stage	0	113	39.37	57	38.26	56	40.58	0.35
	1	56	19.51	26	17.45	30	21.74	
	2	105	36.59	59	39.60	46	33.33	
	3	3	1.05	0	0	3	2.26	
Clinical stage	1	9	3.14	2	1.34	7	5.07	0.30
	2	23	8.01	12	8.05	11	7.97	
	3	48	16.72	21	14.09	27	19.57	
	4	207	72.13	114	76.51	93	67.39	
Tumor grade	1	25	8.71	11	7.38	14	10.14	0.20
	2	168	58.54	82	55.03	86	62.32	
	3	75	26.13	47	31.54	28	20.29	
	4	2	0.70	2	1.34	0	0	
Survival time (month)		21.77		20.73		23.62		0.32
Vital status	Death	110	38.33	57	38.26	53	38.41	0.12
	Alive	177	61.67	92	61.74	85	61.59	

* The difference between the test set and training set was calculated in terms of clinical pathologic factors. Specifically, age was compared with Wilcoxon rank-sum test; gender, clinical T, N, stage, and grade were compared with the chi-squared test; survival time and the status difference were assessed with the log-rank test.

### Statistical Analysis

In the work, the ROC curve was performed via the R package survivalROC (Heagerty et al., 2013), and the area under the ROC curve (AUC) was used to assess the overall performance of radiosensitivity prediction. Kaplan–Meier survival curves were used to further evaluate the significance difference of OS between different groups. A two-tailed *P*-value (*P*) < 0.05 was considered statistically significant in all analyses. The nomogram and the calibration plot were established using R package rms (Harrell et al., 2019) and were used to predict OS of HNSCC patients with RT.

## Results and Discussion

### Identification of a 12-Gene Signature

Based on the gene expression data from 287 patients with RT and 189 patients without RT, 122 DEGs with |log_2_ fold change| ≥ 1 and FDR < 0.01 were obtained. The gene expression, clinical, and CNV/SNV data of these DEGs on the training set were imported into 2SR model. With the probability related to OS should be >80% of HNSCC patients receiving RT, 12 genes were picked out as the signature genes. Next, we calculated the coefficients of these signature genes using multivariable Cox regression model. Finally, in order to predict the radiosensitivity of HNSCC patients, we constructed a gene signature on the training set according to the expression levels of these 12 genes as follows: gene score = *TDRD9* × 6.950E-6 + *CELF3* × 1.106E-2 + *FGF19* × 1.937E-5 + *KCNB2* × 5.388E-3 + *CLDN6* × 1.334E-4 − *BEST2* × 6.053E-4 − *DDX25* × 1.802E-3 − *TMPRSS15* × 4.378E-4 − *ALPI* × 2.134E-3 − *FABP7* × 1.754E-3 − *IL17REL* × 2.132E-3 − *RORB* × 1.182E-3. The details of these 12 signature genes were shown in Supplementary Table S1. Then, based on the gene scores, the patients were divided into the high- and low-score groups, where the cutoff value of -0.06338 was derived from the median score of gene scores on the training set samples. Specifically, on the training (149 HNSCC patients receiving RT) and test (139 patients receiving RT) sets, HNSCC patients with a gene score ≥-0.06338 were divided into the high-score group, while those with a gene score <-0.06338 were divided into the low-score group.

### Radiosensitivity Prediction by the 12-Gene Signature

**FIGURE 2:**
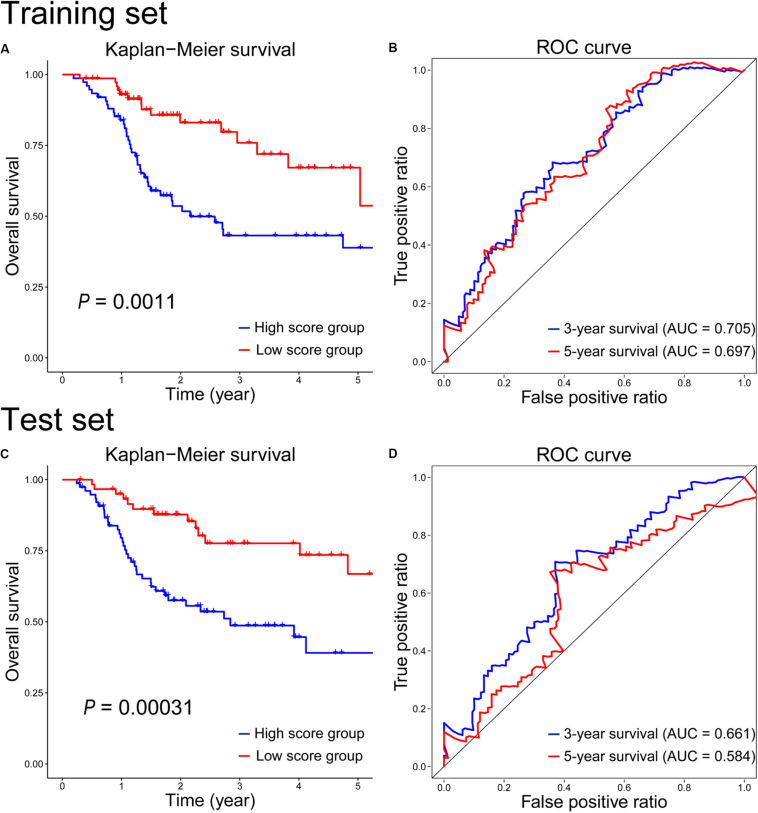
Kaplan–Meier survival and time-dependent ROC curves on the training **(A,B)** and test **(C,D)** sets according to the 12-gene signature.

To assess the radiosensitivity prediction ability of the 12-gene signature on the HNSCC patients, Kaplan–Meier survival and ROC curves were performed on the training and test sets, respectively. On the training set, there was a significant difference on radiosensitivity between the high- and low-score groups (*P* = 0.0011, Figure 2A). As we can see, patients in the high-score group were associated with poor radiosensitivity, while patients in the low-score group showed good radiosensitivity. In the light of the time-dependent ROC curves of 3-year and 5-year survival (Figure 2B), the survival time prediction accuracy of the 12-gene signature for HNSCC patients had AUC of 0.705 at 3 years and 0.697 at 5 years. Furthermore, the prediction performance of the 12-gene signature was evaluated on the test set. As seen in Figure 2C, the survival rate was significantly higher in the low-score group than that in the high-score group (*P* = 0.00031), which was similar with the result of the training set. And the 3-year and 5-year prediction accuracy achieved 0.661 and 0.584, respectively (Figure 2D). The performance on the test set was similar with that on the training set. It indicated the generalization ability of the 12-gene signature was good, and this gene signature could provide a method to predict the radiosensitivity for HNSCC patients. However, the radiosensitivity prediction accuracy of 5-year survival on the test set was lower than that on the training set (Figures 2B,D). There were two possible reasons to explain the causes of the difference. First, the follow-up times were relatively short for HNSCC cohort in TCGA, and the OS of most patients was also less than 5 years. Second, the intrinsic genetic heterogeneity of the tumor could lead to different OS in HNSCC patients with same therapeutic method. The longer the OS, the larger the effect of the intrinsic genetic heterogeneity, and the more difficultly we evaluated this effect with RT.

### Assessment of the 12-Gene Signature in All HNSCC Patients

**FIGURE 3:**
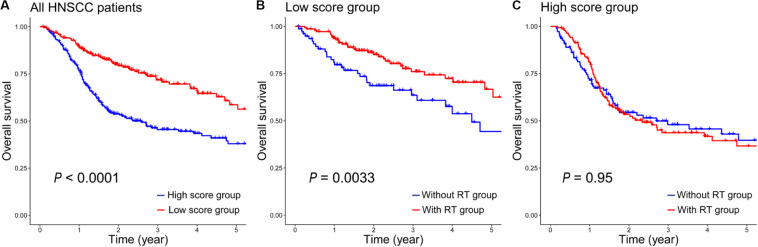
The prognostic values of the 12-gene signature in all HNSCC patients. **(A)** Kaplan–Meier analysis of overall survival in 476 patients according to the 12-gene signature. **(B)** Kaplan–Meier survival curves of patients with/without RT in the low-score group. **(C)** Kaplan–Meier survival curves of patients with/without RT in the high-score group.

Because of the complexity and heterogeneity of tumors, some HNSCC patients are treated by RT with good outcomes, and some patients may have a resistance to RT, even get worse. Therefore, the 12-gene signature is important for the radiosensitivity prediction of HNSCC patients to improve RT options. In addition, we also assessed the prognostic value of the 12-gene signature in a total of 476 HNSCC patients. As seen in Figure 3A, it indicated that patients in the low-score group generally had a higher 5-year survival rate than that in the high-score group (*P* < 0.0001). And there was a significant difference between patients with RT and without RT in the low-score groups (*P* = 0.0033, Figure 3B); however, in the high-score group, the difference was insignificant (*P* = 0.95, Figure 3C). It suggested that patients in the high-score group might have the radioresistance and did not benefit from RT.

**FIGURE 4:**
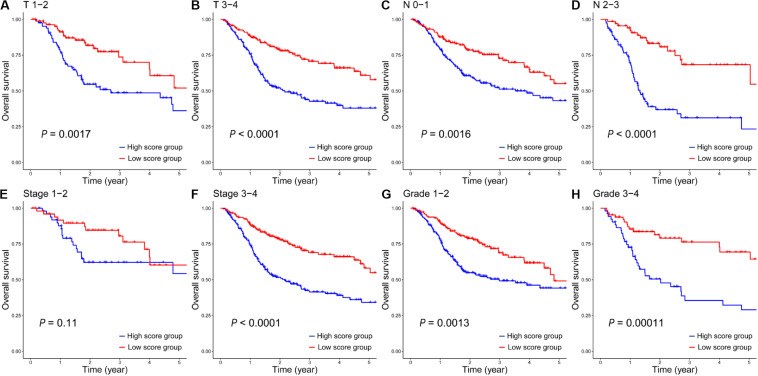
The Kaplan–Meier survival analysis of the 12-gene signature in all HNSCC patients in the high- and low-score groups on clinical subgroups of T 1-2 **(A)**, T 3-4 **(B)**, N 0-1 **(C)**, N 2-3 **(D)**, Stage 1-2 **(E)**, Stage 3-4 **(F)**, Grade 1-2 **(G)** and Grade 3-4 **(H)**.

In addition, based on different clinical variables such as age, gender, T stage, N stage, clinical stage and tumor grade, HNSCC patients were further stratified into different subgroups (Table 1). Then we evaluated the prognostic value of the 12-gene signature on these different subgroups between HNSCC patients in the high- and low-score groups. On the subgroups of clinic T (Figures 4A,B), clinic N (Figures 4C,D), stage 3-4 (Figure 4F), and grade (Figures 4G,H), the survival rates of patients in the low-score group were significantly higher than those in the high-score group. While there was no statistically significant difference of survival rate between patients in the high- and low-score groups in the subgroup of stage 1-2 (Figure 4E). It was mainly due to the small number of patients in this clinical phase, which accounted for only 11% of all HNSCC patients. Furthermore, on the subgroups of age (Supplementary Figures S1A,B) and gender (Supplementary Figure S1C), the survival rates of patients in the low-score group also were significantly longer than those patients in the high-score group. However, the survival rate between female patients (Supplementary Figure S1D) in the high- and low-score groups did not have statistically significant difference, which was similar with that on the stage 1-2 subgroup. Taken together, these results suggested that this 12-gene signature could serve as a novel and reliable biomarker for the radiosensitivity prediction of HNSCC patients.

### Prediction of OS in HNSCC With RT by Nomogram

**FIGURE 5:**
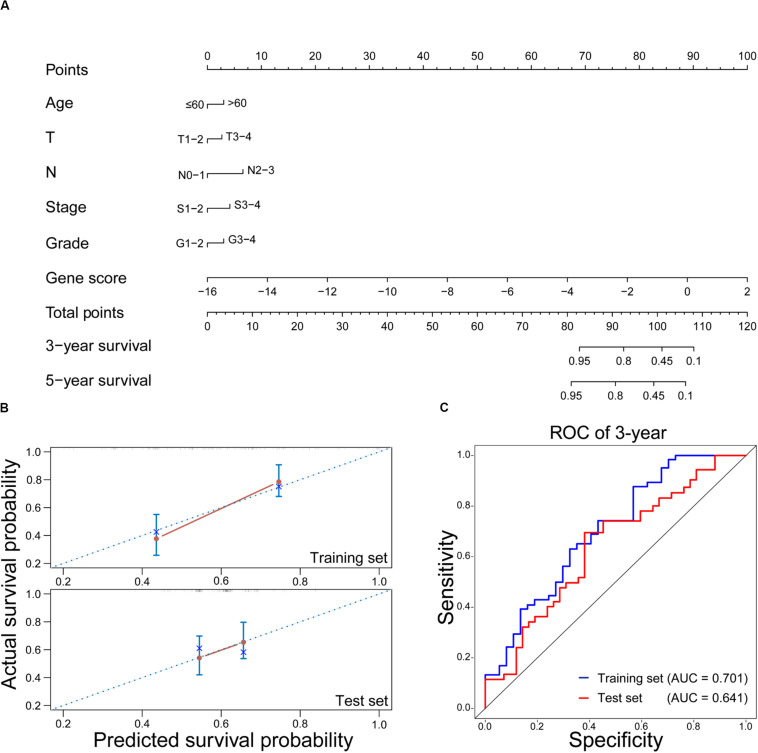
Evaluation of the nomogram on predicting the OS of HNSCC patients with RT. **(A)** Nomogram for predicting the 3-year and 5-year OS in HNSCC with RT. **(B)** Calibration plots of the nomogram on the training and test sets. The 45-degree line represents the real outcomes. **(C)** Time-dependent ROC curves of 3-year OS prediction using the nomogram on the training and test sets.

As a visual tool, nomogram has been widely used in predicting the prognosis of cancers (Lubsen et al., 1978; Gorlia et al., 2008). In this work, the nomogram was used to construct the OS prediction model for HNSCC patients with RT. As shown in Figure 5A, age, T stage, N stage, clinical stage, tumor grade, and gene score were considered as relevant variables for the nomogram construction. Since the points of male and female in the nomogram were similar and closed to zero, gender was not shown here. In addition, the contribution of gene score was very important, and it played a crucial role in survival estimation of HNSCC patients with RT. In order to evaluate the predicted outcomes of nomogram, the calibration plots on the training and test sets were exhibited in Figure 5B. It was found that the predicted results of the nomogram showed good agreements with the actual situations, especially on the test set. Furthermore, according to the time-dependent ROC curves (Figure 5C), the nomogram achieved 0.701 and 0.641 of AUC for 3-year OS on the training and test sets, respectively. It revealed that the nomogram could be used as a promising tool to predict the OS of HNSCC patients with RT.

### Comparison With the Gene Signatures Based on Single Omics Data

Besides the 12-gene signature established using multiple omics data, we also assessed the radiosensitivity prediction ability of the gene signatures based on single omics data, such as gene expression data or CNV/SNV data. Since the 2SR model requires multiple omics data as the input files, we used differential expression analysis, univariable Cox proportional hazards regression analysis (David, 1972), classical LASSO regression model (Tibshirani, 1996, 1997; Segal, 2006), and multivariate Cox regression model to select the most important biomarkers and take their linear combination as a predictor of radiosensitivity based on single omics data. Firstly, differential expression analysis was used to identify DEGs by analyzing gene expression profiles of HNSCC patients with RT and without RT. Of note, the construction of the gene signature based on the CNV/SNV data did not use differential expression analysis. Secondly, univariate Cox proportional hazards regression analysis and LASSO logistic regression model were used to screen out the characteristic genes associated with survival. Thirdly, multivariate Cox regression model was used to establish a gene signature for radiosensitivity prediction. Then, HNSCC patients receiving RT were divided into the high- and low-score groups according to the median score on the training set patients. Finally, Kaplan–Meier survival analysis and ROC curves were conducted to observe the difference of survival rate between the patients in the high- and low-score groups.

**FIGURE 6:**
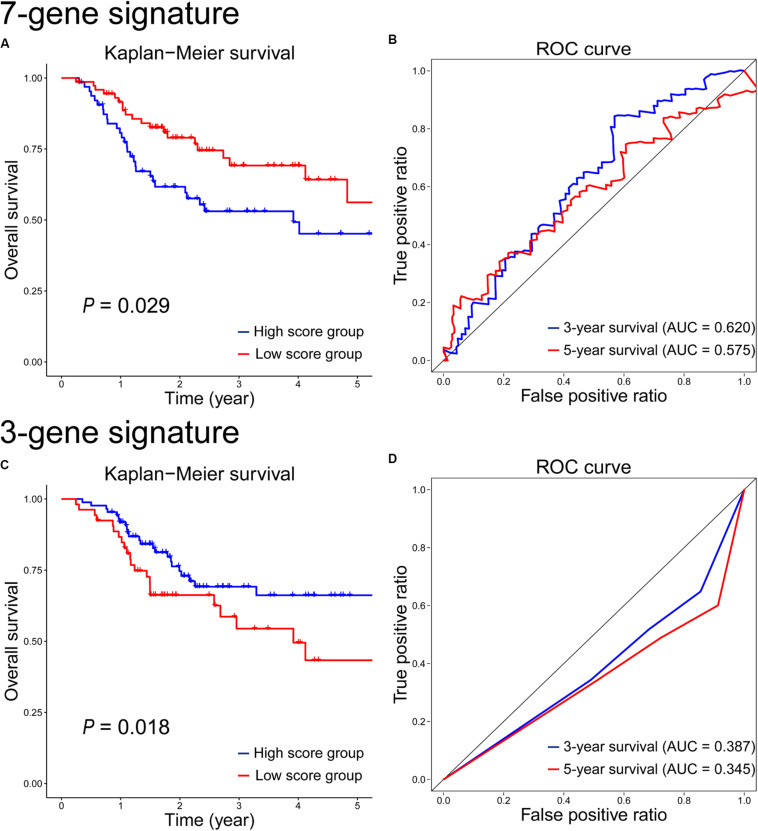
Kaplan–Meier survival and time-dependent ROC curves on the test set according to the 7-gene signature **(A,B)** and the 3-gene signature **(C,D)**.

Based on gene expression data, a 7-gene signature was constructed for radiosensitivity prediction, and the gene score = *CHGB* × 1.121E-4 + *ODAM* × 3.603E-5 + *RP11-169K17.3* × 8.518E-2 − *ZNF541* × 8.229E-5 − *CLGN* × 2.952E-3 − *AC011747.3* × 1.407E-1 − *RP11-203B7.2* × 1.357E-1. The details of these 7 signature genes were shown in Supplementary Table S2. The survival analysis results on the test set were shown in Figure 6A. Obviously, the difference between the high- and low-score groups was significant (*P* = 0.029), which was similar with that on the training set (*P* = 0.011, Supplementary Figure S2A). The prognostic accuracy of the 7-gene signature for HNSCC patients receiving RT was 0.62 at 3 years and 0.575 at 5 years (Figure 6B), which were both lower than the performances of the 12-gene signature based on multiple omics data.

Based on CNV/SNV data, a 3-gene signature was developed for radiosensitivity prediction, and the gene score = − *BCLAF1* × 8.084E-1 − *ABCB9* × 3.330E-1 − *MIS18BP1* × 3.697E-1. The details of the 3-gene signature were shown in Supplementary Table S3. The survival analysis on the test set showed that there was a significant difference between the high- and low-score groups (*P* = 0.018, Figure 6C), but it was inconsistent with the result on the training set (*P* = 0.0068, Supplementary Figure S2C). The 3-year and 5-year survival prognostic accuracy of the 3-gene signature were 0.387 and 0.345 on the test set, respectively (Figure 6D), which were far less than the performances of the 12-gene signature using multiple omics data. As single omics data, the sample-by-gene matrix based on CNV/SNV data was sparse, and the genetic information extracted from the sparse matrix was very limited. So the radiosensitivity prediction accuracy of the gene signature based on CNV/SNV data was not good.

At first, we compared the 12-gene signature with those gene signatures (7-gene and 3-gene signatures) based on single omics data. The results showed that the 12-gene signature achieved the highest separation ability, and it significantly stratified patients into the low- and high-score groups on the test set (*P* = 0.00031 vs. *P* = 0.029 vs. *P* = 0.018). It also had the highest accuracy of survival estimation among these gene signatures (3-year survival: 0.661 vs. 0.620 vs. 0.387; 5-year survival: 0.584 vs. 0.575 vs. 0.345) on the test set. In addition, we performed GO and KEGG enrichment analyses, and the result showed there were no significant enrichments. Moreover, on the GO and KEGG terms (Jiao et al., 2012; Xi et al., 2020), there are also no correlations between these signature genes and radiation. Maybe these genes were novel candidate targets and biomarkers correlated with radiation. However, given the performance of 12-gene signature was better than those of 7-gene and 3-gene signatures, the genes in 12-gene signature were more important on radiation response and radiosensitivity prediction. Nevertheless, their roles need to be proved by biological and clinical experiments. Furthermore, we evaluated the performance of the 5-miRNA signature (Chen et al., 2018) on the test set. As shown in Supplementary Figure S3, there is no significant different between the high- and low-score groups based on 5-miRNA signature, and the items of AUC (0.493 and 0.450) on 3-year and 5-year survivals were lower than those (0.661 and 0.584) based on 12-gene signature. In a word, the 12-gene signature based on multiple omics data was a relatively reliable biomarker to predict whether the HNSCC patient benefit from the treatment of RT.

## Conclusion

In this study, we used the gene expression, clinical, and CNV/SNV data to develop and validate the 12-gene signature, which may serve as a promising prognostic biomarker for the radiosensitivity prediction of HNSCC patients. Furthermore, we constructed a nomogram based on gene score and clinical variables, which might be a useful tool on the survival estimation of HNSCC patients receiving RT. Finally, we systemically compared the prognosis ability of the gene signatures based on multiple and single omics data, and the results showed that the 12-gene signature based on multiple omics data was more accurate in predicting radiotherapy response and survival rate of HNSCC patients. However, this study also has some limitations. First, these signature genes as biomarkers for radiosensitivity in HNSCC deserve further biological and clinical verification. Second, gene expression signatures are subject to sampling bias caused by the complexity and heterogeneity of tumors, so we will consider the subtypes of tumor in the future study.

## Data Availability Statement

All datasets presented in this study are included in the article/Supplementary Material.

## Author Contributions

JX, LW, GZ, MH, JL, and ZY conceived and designed the study. MH and JL performed the experiments. MH, JL, and CD wrote the manuscript. JX, YB, and PW reviewed the manuscript. All authors read and approved the final version of this manuscript.

## Conflict of Interest

The authors declare that the research was conducted in the absence of any commercial or financial relationships that could be construed as a potential conflict of interest.

## Supplementary Material

FIGURE S1 The Kaplan-Meier survival analysis of the 12-gene signature in all HNSCC patients in the high and low score groups on age ≤ 60, age > 60, male, female subgroups.

FIGURE S2 Kaplan-Meier survival and time-dependent ROC curves on the training set according to the 7-gene signature and the 3-gene signature.

TABLE S1 The information of genes in the 12-gene signature.

TABLE S2 The information of genes in the 7-gene signature.

TABLE S3 The information of genes in the 3-gene signature.

## References

[B1] Alsahafi E. Begg K. Amelio I. Raulf N. Lucarelli P. Sauter T. (2019). Clinical update on head and neck cancer: molecular biology and ongoing challenges. *Cell Death Dis.* 10:540 10.1038/s41419-019-1769-1769 PMC6629629 31308358

[B2] Benner A. Zucknick M. Hielscher T. Ittrich C. Mansmann U. (2010). High-dimensional Cox models: the choice of penalty as part of the model building process. *Biomed. J.* 52 50–69. 10.1002/bimj.200900064 20166132

[B3] Chakraborty S. Hosen M. I. Ahmed M. Shekhar H. U. (2018). Onco-multi-OMICS approach: a new frontier in cancer research. *Biomed Res. Int.* 2018:9836256. 10.1155/2018/9836256 30402498 PMC6192166

[B4] Chen L. Wen Y. Zhang J. Sun W. Lui V. W. Y. Wei Y. (2018). Prediction of radiotherapy response with a 5-microRNA signature-based nomogram in head and neck squamous cell carcinoma. *Cancer Med.* 7 726–735. 10.1002/cam4.1369 29473326 PMC5852342

[B5] David C. R. (1972). Regression models and life tables. *J. R. Stat. Soc. B.* 34 187–220.

[B6] Eschrich S. Zhang H. L. Zhao H. Y. Boulware D. Lee J. H. Bloom G. (2009). Systems biology modeling of the radiation sensitivity network: a biomarker discovery platform. *Int. J. Radiat. Oncol. Biol. Phys.* 75 497–505. 10.1016/j.ijrobp.2009.05.056 19735874 PMC2762403

[B7] Goldman M. Craft B. Hastie M. Repeèka K. McDade F. Kamath A. (2019). The UCSC Xena platform for public and private cancer genomics data visualization and interpretation. *bioRxiv [Preprint]* Available online at: https://www.biorxiv.org/content/10.1101/326470v6 (accessed September 26, 2019).

[B8] Gorlia T. van den Bent M. J. Hegi M. E. Mirimanoff R. O. Weller M. Cairncross J. G. (2008). Nomograms for predicting survival of patients with newly diagnosed glioblastoma: prognostic factor analysis of EORTC and NCIC trial 26981-22981/CE.3. *Lancet Oncol.* 9 29–38. 10.1016/S1470-2045(07)70384-70384 18082451

[B9] Gui J. Li H. (2005). Penalized Cox regression analysis in the high-dimensional and low-sample size settings, with applications to microarray gene expression data. *Bioinformatics* 21 3001–3008. 10.1093/bioinformatics/bti422 15814556

[B10] Han P. Ji X. Zhang M. Gao L. J. E. R. M. P. S. (2018). Upregulation of lncRNA LINC00473 promotes radioresistance of HNSCC cells through activating Wnt/beta-catenin signaling pathway. *Eur. Rev. Med. Pharmacol.* 22 7305–7313. 10.26355/eurrev_201811_16267 30468475

[B11] Harrell F. E. Jr. Harrell M. F. E. Jr. Hmisc D. (2019). *Package ‘rms’.* Nashville: Vanderbilt University, 229.

[B12] Heagerty P. J. Lumley T. Pepe M. S. (2000). Time-dependent ROC curves for censored survival data and a diagnostic marker. *Biometrics* 56 337–344. 10877287 10.1111/j.0006-341x.2000.00337.x

[B13] Heagerty P. J. Saha-Chaudhuri P. Saha-Chaudhuri M. P. (2013). *Package ‘survivalROC’.*

[B14] Hu X. H. Xie W. B. Wu C. C. Xu S. Z. (2019). A directed learning strategy integrating multiple omic data improves genomic prediction. *Plant Biotechnol. J.* 17 2011–2020. 30950198 10.1111/pbi.13117 PMC6737184

[B15] Jemal A. Bray F. Center M. M. Ferlay J. Ward E. Forman D. (2011). Global cancer statistics. *CA Cancer J. Clin.* 61 69–90. 10.3322/caac.20107 21296855

[B16] Jiao X. Sherman B. T. Huang D. W. Stephens R. Baseler M. W. Lane H. C. (2012). DAVID-WS: a stateful web service to facilitate gene/protein list analysis. *Bioinformatics* 28 1805–1806. 22543366 10.1093/bioinformatics/bts251 PMC3381967

[B17] Kamarudin A. N. Cox T. Kolamunnage-Dona R. (2017). Time-dependent ROC curve analysis in medical research: current methods and applications. *BMC Med. Res. Methodol.* 17:53. 10.1186/s12874-017-0332-6 28388943 PMC5384160

[B18] Leucci E. Vendramin R. Spinazzi M. Laurette P. Fiers M. Wouters J. (2016). Melanoma addiction to the long non-coding RNA SAMMSON. *Nature* 531 518–522. 27008969 10.1038/nature17161

[B19] Lin W. Feng R. Li H. Z. (2015). Regularization methods for high-dimensional instrumental variables regression with an application to genetical genomics. *J. Am. Stat. Assoc.* 110 270–288. 10.1080/01621459.2014.908125 26392642 PMC4573639

[B20] Love M. I. Huber W. Anders S. (2014). Moderated estimation of fold change and dispersion for RNA-seq data with DESeq2. *Genome Biol.* 15:550 10.1186/s13059-014-0550-558 PMC4302049 25516281

[B21] Lubsen J. Pool J. Der Does E. V. (1978). A practical device for the application of a diagnostic or prognostic function. *Methods Inf. Med.* 17 127–129. 10.1055/s-0038-1636613 661607

[B22] Ma J. B. Li R. Wang J. (2019). Characterization of a prognostic four-gene methylation signature associated with radiotherapy for head and neck squamous cell carcinoma. *Mol. Med. Report.* 20 622–632. 10.3892/mmr.2019.10294 31180552 PMC6579992

[B23] Scaife J. E. Barnett G. C. Noble D. J. Jena R. Thomas S. J. West C. M. L. (2015). Exploiting biological and physical determinants of radiotherapy toxicity to individualize treatment. *Brit. J. Radiol.* 88:20150172. 10.1259/bjr.20150172 26084351 PMC4628540

[B24] Segal M. R. (2006). Microarray gene expression data with linked survival phenotypes: diffuse large-B-cell lymphoma revisited. *Biostatistics* 7 268–285. 10.1093/biostatistics/kxj006 16284340

[B25] Suh Y. E. Raulf N. Gaken J. Lawler K. Urbano T. G. Bullenkamp J. (2015). MicroRNA-196a promotes an oncogenic effect in head and neck cancer cells by suppressing annexin A1 and enhancing radioresistance. *Int. J. Cancer* 137 1021–1034. 10.1002/ijc.29397 25523631

[B26] Tibshirani R. (1996). Regression shrinkage and selection via the lasso. *J. R. Stat. Soc. B.* 58 267–288. 10.1111/j.2517-6161.1996.tb02080.x

[B27] Tibshirani R. (1997). The lasso method for variable selection in the Cox model. *Stat. Med.* 16 385–395. 10.1002/(sici)1097-0258(19970228)16:4<385::aid-sim380<3.0.co;2-3 9044528

[B28] Tomczak K. Czerwinska P. Wiznerowicz M. (2015). The Cancer Genome Atlas (TCGA): an immeasurable source of knowledge. *Contemp. Oncol.* 19 A68–A77. 10.5114/wo.2014 PMC4322527 25691825

[B29] Tripepi G. Catalano F. (2004). Kaplan-Meier analysis. *G. Ital. Nefrol.* 21 540–546. 15593021

[B30] van der Heijden M. Essers P. B. M. de Jong M. C. de Roest R. H. Sanduleanu S. Verhagen C. V. M. (2019). Biological determinants of chemo-radiotherapy response in HPV-negative head and neck cancer: a multicentric external validation. *Front. Oncol.* 9:1470. 10.3389/fonc.2019.01470 31998639 PMC6966332

[B31] Wang H. C. Chan L. P. Cho S. F. (2019). Targeting the immune microenvironment in the treatment of head and neck squamous cell carcinoma. *Front. Oncol.* 9:1084. 10.3389/fonc.2019.01084 31681613 PMC6803444

[B32] Wang W. Zhou Y. Cheng M.-T. Wang Y. Zheng C.-H. Xiong Y. (2020). Potential pathogenic genes prioritization based on protein domain interaction network analysis. *IEEE/ACM Trans. Comput. Biol. Bioinform.* 99 1–1. 10.1109/TCBB.2020.2983894 32248121

[B33] Weng J. H. Yu C. C. Lee Y. C. Lin C. W. Chang W. W. Kuo Y. L. (2016). miR-494-3p induces cellular senescence and enhances radiosensitivity in human oral squamous carcinoma cells. *Int. J. Mol. Sci.* 17:1092. 10.3390/ijms17071092 27399693 PMC4964468

[B34] Xi J. Li A. Wang M. (2018). HetRCNA: a novel method to identify recurrent copy number alternations from heterogeneous tumor samples based on matrix decomposition framework. *IEEE/ACM Trans. Comput. Biol. Bioinform.* 17 422–434. 29994262 10.1109/TCBB.2018.2846599

[B35] Xi J. Yuan X. Wang M. Li A. Li X. Huang Q. J. B. (2020). Inferring subgroup-specific driver genes from heterogeneous cancer samples via subspace learning with subgroup indication. 36 1855–1863. 10.1093/bioinformatics/btz793 31626284

