# Supplementary material for: Prediction of Radiosensitivity in Head and Neck Squamous Cell Carcinoma Based on Multiple Omics Data
Source: Front Genet. 2020 Aug 18;11:960. doi: 10.3389/fgene.2020.00960 (PMC7461877; doi:10.3389/fgene.2020.00960)
Supplement: FIGURE S1 — The Kaplan-Meier survival analysis of the 12-gene signature in all HNSCC patients in the high and low score groups on age ≤ 60, age > 60, male, female subgroups. [file Data_Sheet_1.docx]

Supplementary Material

# Supplementary Tables

**Supplementary Table 1.** The information of genes in the 12-gene signature

| **Ensemble ID** | **Gene symbol** | **Gene type** | **Coefficients** | **Description** |
| --- | --- | --- | --- | --- |
| ENSG00000039987.5 | *BEST2* | Protein_coding | -6.053E-4 | Bestrophin 2 |
| ENSG00000154646.7 | *TMPRSS15* | Protein_coding | -4.378E-4 | Transmembrane serine protease 15 |
| ENSG00000162344.3 | *FGF19* | Protein_coding | 1.937E-5 | Fibroblast growth factor 19 |
| ENSG00000163295.4 | *ALPI* | Protein_coding | -2.134E-3 | Alkaline phosphatase, intestinal |
| ENSG00000182674.5 | *KCNB2* | Protein_coding | 5.388E-3 | Potassium voltage-gated channel subfamily B member 2 |
| ENSG00000184697.6 | *CLDN6* | Protein_coding | 1.334E-4 | Claudin 6 |
| ENSG00000188263.9 | *IL17REL* | Protein_coding | -2.132E-3 | Interleukin 17 receptor E like |
| ENSG00000198963.9 | *RORB* | Protein_coding | -1.182E-3 | RAR related orphan receptor B |
| ENSG00000109832.11 | *DDX25* | Protein_coding | -1.802E-3 | DEAD-box helicase 25 |
| ENSG00000156414.17 | *TDRD9* | Protein_coding | 6.950E-6 | Tudor domain containing 9 |
| ENSG00000159409.13 | *CELF3* | Protein_coding | 1.106E-2 | CUGBP elav-like family member 3 |
| ENSG00000164434.10 | *FABP7* | Protein_coding | -1.754E-3 | Fatty acid binding protein 7 |

**Supplementary Table 2.** The information of genes in the 7-gene signature

| **Ensemble ID** | **Gene symbol** | **Gene type** | **Coefficients** | **Description** |
| --- | --- | --- | --- | --- |
| ENSG00000089199.8 | *CHGB* | Protein_coding | 1.121E-4 | Chromogranin B |
| ENSG00000109205.15 | *ODAM* | Protein_coding | 3.603E-5 | Odontogenic, ameloblast associated |
| ENSG00000118156.11 | *ZNF541* | Protein_coding | -8.229E-5 | Zinc finger protein 541 |
| ENSG00000153132.11 | *CLGN* | Protein_coding | -2.952E-3 | Calmegin |
| ENSG00000231435.1 | *AC011747.3* | lncRNA | -1.407E-1 | - |
| ENSG00000260303.1 | *RP11-203B7.2* | lncRNA | -1.357E-1 | - |
| ENSG00000272483.1 | *RP11-169K17.3* | lncRNA | 8.518E-2 | - |

**Supplementary Table 3.** The information of genes in the 3-gene signature

| **Ensemble ID** | **Gene symbol** | **Gene type** | **Coefficients** | **Description** |
| --- | --- | --- | --- | --- |
| ENSG00000029363.14 | *BCLAF1* | Protein_coding | -8.084E-1 | BCL2 associated transcription factor 1 |
| ENSG00000150967.16 | *ABCB9* | Protein_coding | -3.330E-1 | ATP binding cassette subfamily B member 9 |
| ENSG00000129534.12 | *MIS18BP1* | Protein_coding | -3.697E-1 | MIS18 binding protein 1 |

# Supplementary Figures


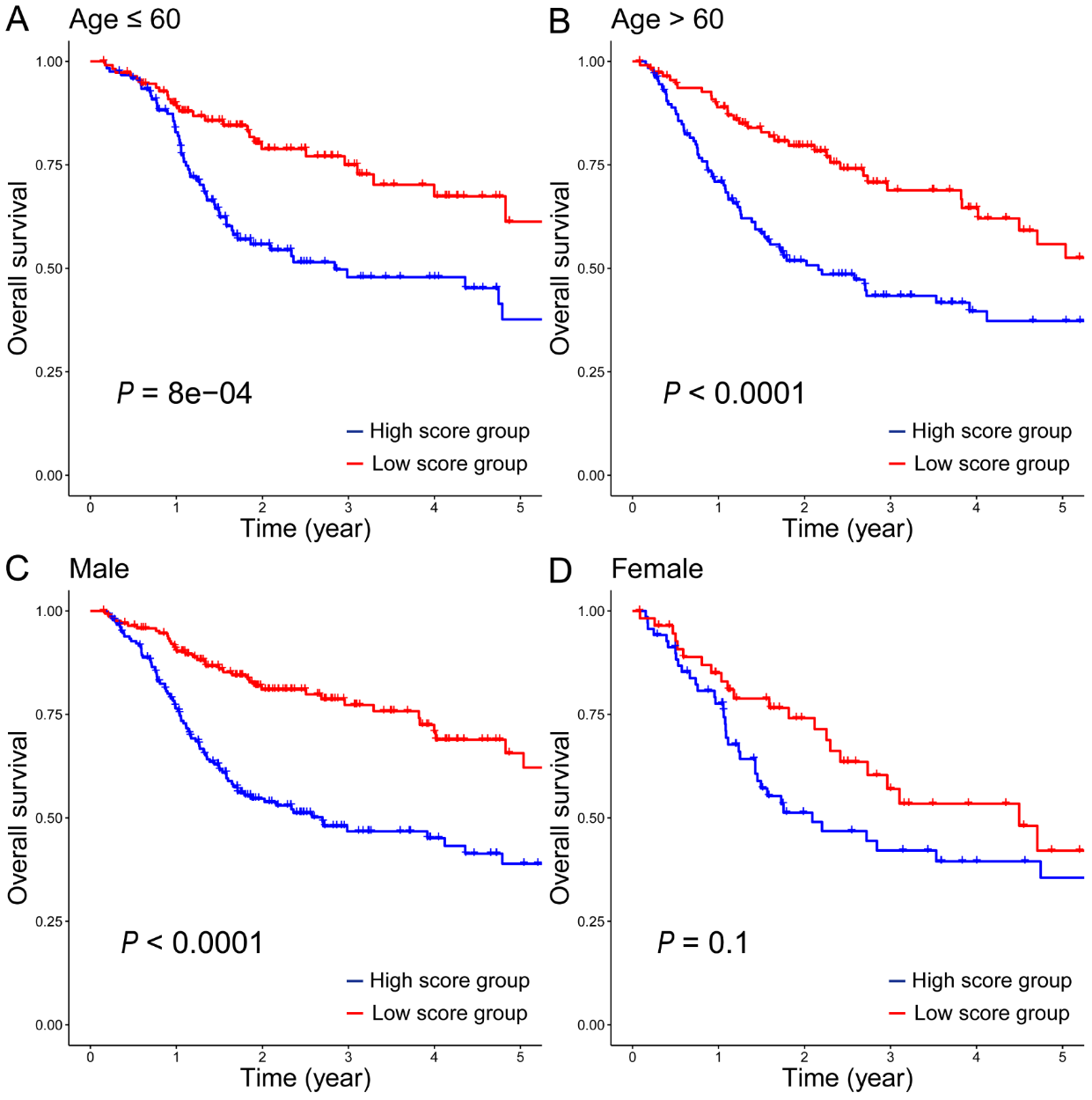


**Supplementary Figure 1.** The Kaplan-Meier survival analysis of the 12-gene signature in all HNSCC patients in the high and low score groups on age ≤ 60 (**A**), age > 60 (**B**), male (**C**), female (**D**) subgroups.


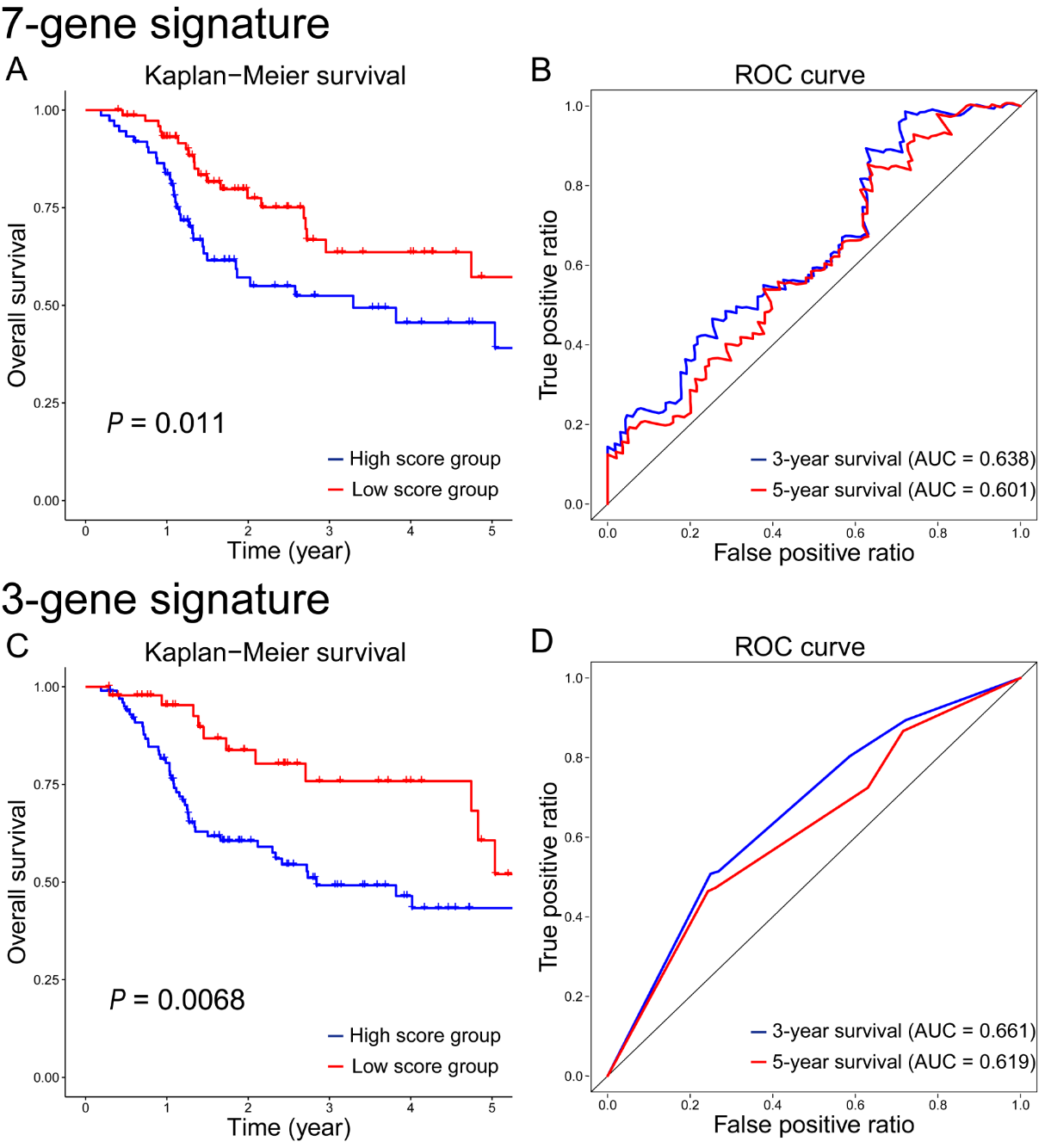


**Supplementary Figure 2.** Kaplan-Meier survival and time-dependent ROC curves on the training set according to the 7-gene signature (**A** and **B**) and the 3-gene signature (**C** and **D**).


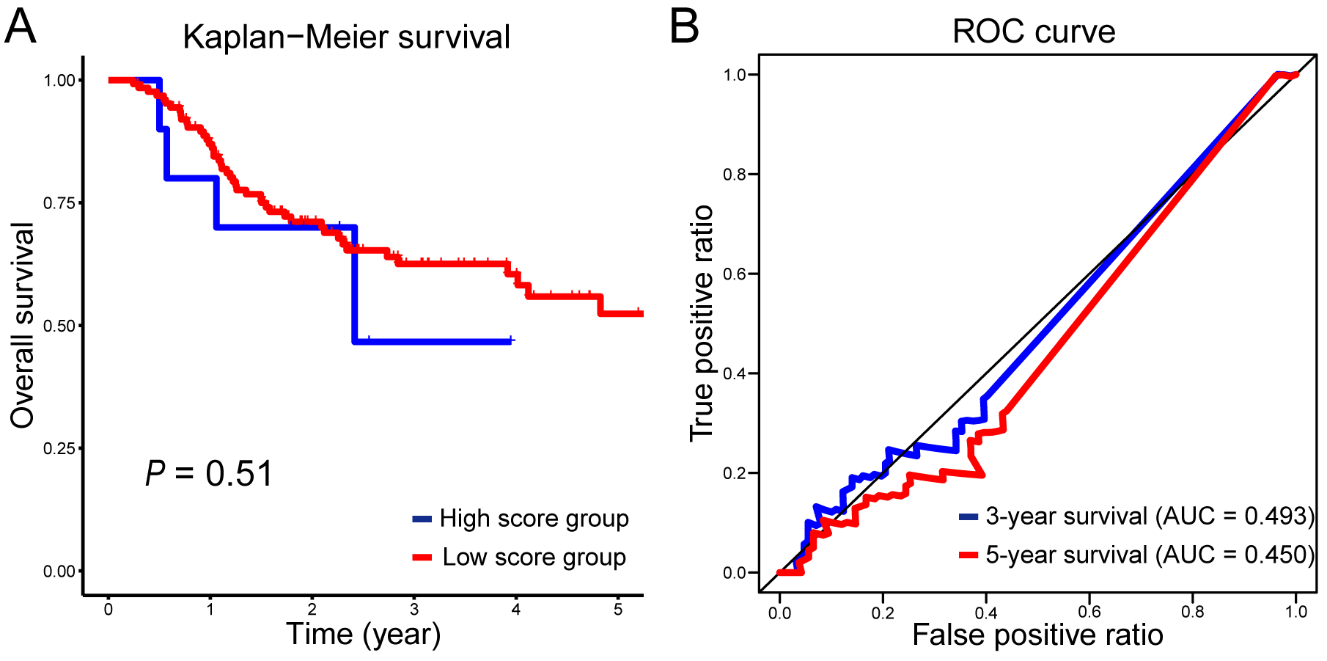


**Supplementary Figure 3.** Kaplan-Meier survival (**A)** and time-dependent ROC (**B)** curves on the test set according to the 5-miRNA signature.
